# Influence of Ultra-Heat Treatment on Properties of Milk Proteins

**DOI:** 10.3390/polym13183164

**Published:** 2021-09-18

**Authors:** Thummalacharla Chaitanya Krishna, Agnieszka Najda, Aarti Bains, Mansuri M. Tosif, Rafał Papliński, Magdalena Kapłan, Prince Chawla

**Affiliations:** 1 Department of Food Technology and Nutrition, Lovely Professional University, Phagwara, Punjab 144411, India; chaitanyakrish1998@gmail.com (T.C.K.); tosifmansuri444@gmail.com (M.M.T.); 2 Department of Vegetable Crops and Medicinal Plants, University of Life Science in Lublin, Doświadczalna Street 51A, 20-280 Lublin, Poland; rafal.paplinski@up.lublin.pl; 3 Department of Biotechnology, CT Institute of Pharmaceutical Sciences, South Campus, Jalandhar, Punjab 144020, India; aarti05888@gmail.com; 4 Department of Pomology, Nursery, and Enology, University of Life Sciences in Lublin, 20-033 Lublin, Poland; magdalena.kaplan@up.lublin.pl

**Keywords:** milk, ultra-high temperature treatment, casein, whey protein, immunoglobulin

## Abstract

Milk can be considered one of the primary sources of nutrients for the mammalian neonate. Therefore, milk and milk-based products, such as infant formula, whey protein isolate, different varieties of cheese, and others are prepared to meet the nutritional requirements of the consumer. Due to its significant nutritional components and perishable nature, a variety of pathogenic microorganisms can grow and multiply quickly in milk. Therefore, various heat treatments can be employed for the improvement of the shelf life of milk. In comparison to pasteurized milk, due to excessive and severe heating, UHT milk has a more cooked flavor. During storage, changes in the physicochemical properties of milk can lead to off-flavors, undesirable browning, separation of fat, sediment formation, or gelation during the subsequent storage. Several important factors such as processing parameters, time-temperature abuse (storage condition), and packaging type also influence the quality characteristics and consumer acceptance of the milk; however, the influence of heat treatments on milk protein is inconstant. The major protein modifications that occur during UHT treatment are denaturation and aggregation of the protein, and chemical modifications of its amino acids. These UHT-induced protein alterations can change digestibility and the overall biological influence of the intake of these proteins. Therefore, this review is focused on the influence of UHT on the physicochemical and structural attributes of milk proteins during storage. There are many indications of milk proteins present in the UHT milk, and milk products are altered during processing and storage.

## 1. Introduction

Milk is a complex biological fluid, produced in the mammary gland of female mammalian species. It is produced to feed the neonate from birth to weaning [1]. It is a highly nutritious and readily digested food, i.e., rich in minerals, proteins, and energy in an aqueous solution. This provides the neonate with essential amino acids and many other important compounds such as hormones, growth factors, and protective agents [2].

Furthermore, milk provides quality proteins (casein and whey proteins) that cannot be obtained from any other food material [3]. Milk proteins also exhibit a high range of positive health and therapeutic benefits, such as aiding muscle growth, promoting fat loss, and strengthening bones in both adults and children. Humans consume cow milk primarily; however, globally, people obtain milk from many other animals, including buffalo, yaks, goats, sheep, reindeer, and camels. Generally, cow’s milk contains 80% of casein and 20% of whey protein, and both of the proteins always provide health benefits and are sources of a wide range of peptides with potential bioactive properties [4,5]. The function of milk protein is to supply young mammals with the essential amino acids that are required for the growth and development of the muscular body [5]. For the past centuries, humans have used milk and dairy-derived products to supplement their diet, and these dairy products are still a major food source. Furthermore, the physicochemical properties and textural properties of various dairy products is dependent upon the functional properties of the milk proteins [6,7]. The properties of the milk proteins also influence the quality of milk and milk-based products during various processing and storage conditions. However, due to the perishable nature of milk, research efforts are focused on the investigation of different processes to improve the shelf-life of milk for a longer period. Therefore, severe heat treatments became more popular due to their potential to enhance the shelf-life of the milk without further requirement of refrigeration storage [4]. Ultra-high temperature (UHT) includes the heating of milk at 135–145 °C for 2–3 s, which kills the spore-forming pathogenic microorganism, resulting in a product with a shelf-life of several months when stored at room temperature [6]. Due to the extended shelf-life of UHT-treated milk, it could easily transport to remote areas without refrigeration. In comparison to pasteurized milk, due to excessive and severe heating, UHT milk has more cooked and sulphureous flavors. Additionally, during storage, changes in the physicochemical properties of milk can lead to off-flavors, undesirable browning, separation of fat, sediment formation, or gelation during the subsequent storage [7]. Several important factors such as processing parameters, time-temperature abuse (storage condition), and packaging type also influence the quality characteristics and consumer acceptance. As well, *Psychrotrophs* can grow in refrigerated milk, and the majority of these bacteria produce heat-stable extracellular proteases that remain active after UHT treatment. This gives the final UHT products a bitter flavor, and also leads to gelation [8]. The population of *psychrotrophs* in fresh raw milk obtained from healthy cows is less than 10^2^ colony-forming unit (CFU) mL^−1^ in acceptable hygienic conditions, accounting for 10% of the total microbiota [9]. The ambient temperatures of processing and storage of UHT-processed milk can vary from 0 °C to greater than or equal to 50 °C in cold countries, tropical zones, and some storage facilities [10]. However, these variable conditions significantly influence the proteins of the milk products, which can undergo several changes before the product is consumed. To avoid a declining nutritional value and to ensure the stability of the product, these changes must be understood so that the damage to the product can be minimized [11]. During processing and storage, the changes in proteins are caused by enzymatic activity, physicochemical interactions, and microbiological contamination. Ultra-high temperature processed milk has a shelf life of 6–9 months; however, some companies are claiming a shelf life of 12 months for their products [6,12]. Moreover, there are many ways in which the product can be damaged during storage, even under proper conditions. The legal expiry date for UHT milk in some countries is <90 days [13]. In addition, milk proteins are the most important components of the milk, which are helpful in various biological functions in the human body; however, during storage of UHT milk, aggregates of milk proteins or protein particles of various sizes form that ultimately influence the overall quality of the milk [14]. Aggregation of milk proteins has been shown to increase with variation in storage temperature [15]. Moreover, the aggregation of milk protein is a three-dimensional network that occurs either through enzymatic or non-enzymatic (severe heat) processes. Besides, during storage, changes in the physicochemical properties of milk can lead to off-flavors, undesirable browning, separation of fat, sediment formation, or gelation during the subsequent storage. Several important factors such as processing parameters, storage condition, and packaging type also influence the quality characteristics and consumer acceptance of the milk; however, the influence of heat treatments on milk protein is inconstant. The major protein modifications that occur during UHT treatment are denaturation and aggregation of the protein, and chemical modifications of its amino acids. These UHT-induced protein alterations can change digestibility and the overall biological influence of the intake of these proteins [12,13,14,15]. Therefore, in this review, we discussed the structural chemistry of milk proteins and their fractions. The overall quality of UHT milk and the toxicological and physicochemical changes of UHT-treated milk proteins during processing and storage are elaborately discussed.

## 2. Milk Proteins and Their Fractions

Milk contains approximately 3.5% by weight protein, which is a highly complex system. This milk protein is usually divided into two main fractions based on their solubility nature. Casein proteins are about 75% to 80% of the total protein in the milk and precipitate at pH 4.6 at 20 °C, while 20% of the protein remains in the serum. In the serum, about 15% are whey proteins, which are soluble under the above-mentioned conditions. The rest of the proteins found in milk are trace fractions of glycoproteins [16]. Proteins are made up of a polypeptide chain of amino acid residues joined together by peptide bonds and cross-linked by disulfide bonds. An acid carboxyl group and a weak basic amino group are both joined by a hydrocarbon chain that is unique to each amino acid [17]. However, the casein micelle is spherical and made up of smaller units known as submicelles. Herein, the submicelles vary in composition, consisting of κ-casein and α_s_-casein, or α_s_-casein and β-casein. They are linked together by small calcium phosphate cluster bridges [11]. Moreover, quaternary, secondary, and tertiary structures are all part of the three-dimensional organization of proteins. The three-dimensional arrangement of amino acid residues that are close to each other in the linear sequence is referred to as a secondary structure. Secondary structures rising from regular and periodic steric linkages include the helix and pleated sheet. The tertiary structure refers to the spatial arrangement of amino acid residues that are wide apart in the linear sequence, allowing for more folding and coiling to occur [16]. A protein is considered a globular protein if it is tightly coiled and folded into a spherical shape, and a fibrous protein if it is made up of long peptide chains that are intermolecularly connected. When proteins with two or more polypeptide chain subunits are linked, they form a quaternary structure [12]. The milk proteins include α_s1_-casein, α_s2_-casein, β-casein, κ-casein, α-lactalbumin (αLA), and β-lactoglobulin (βLG), which are the main components of milk and whey products.

### 2.1. Casein and Their Fractions

**Figure 1:**
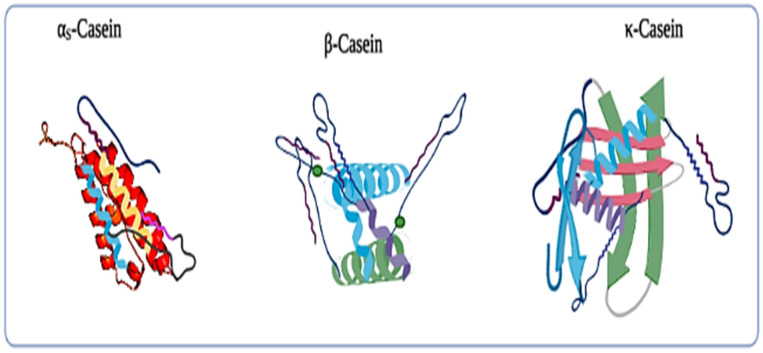
Polymeric structure of different fractions of casein protein. Casein exists in fresh milk in the form of a micelle structure, which is a complex aggregate of proteins (α_s1_-casein, α_s2_-casein, β-casein, κ-casein, and several minor proteins) and colloidal phosphate calcium.

Caseins (heterogeneous group of phosphoproteins) are the main protein group in bovine milk, and it is a key functional protein to the dairy ingredients that are used universally in the food industry. Micellar caseins are the colloidal components that can be explained as a supramolecule system, and it consists of multiple molecular entities remaining together and organized through non-covalent intermolecular binding interactions [2,3,4,5,6,7]. Colloidal casein consists of a mixture of calcium phosphate stabilized by calcium insoluble proteins, and it serves as a prime nutritional source of calcium, phosphate, and amino acids to meet the growth and energy requirements of mammalian neonates. They also have the biological function of transporting calcium phosphate without calcification through the mammary milk system [6,7,8]. Primarily, casein micelle provides fluidity to casein molecules, and solubilize phosphate and calcium. These protein molecules consist of 19–25 kDa molecular weight, and lack high levels of secondary or tertiary protein structures. Furthermore, several prolines exist in β-casein, κ-casein, α_s1_-casein, and α_s2_-casein as 35, 20, 17, and 10 proline residues, respectively, and their properties are shown in Table 1 [14,15,16,17]. Due to the presence of a cyclic amine in its side chain, the proline prevents the formation of α-helices and β-turns in casein structures [18]. Furthermore, casein accounts for about 80% of the total nitrogen content in bovine milk, whereas it only accounts for 40% of the protein in human milk. The white color in the milk is mainly caused by light scattering in casein micelles [19]. At trace levels, the fat globule membrane contains numerous specialized proteins, including many enzymes. These proteins account for 1% of the total protein in milk [16]. Structurally, the casein-micelle is being studied extensively due to its importance in the functional behavior of milk and milk products. However, a large casein-micelle size interferes with absolute structure determination; therefore, to explain the structural chemistry of caseins, different models have been proposed. Models can be classified into three categories, including the coat–core model, the subunit or submicelle model, and the internal structure model. Various reports revealed a detailed description of the casein models, and also explained the internal structure of the casein micelles [12,13,14,15,16,17]. The casein fractions comprise of four principal primary proteins, namely α_s1_-casein, α_s2_-casein, β-casein, and κ- casein, and several minor proteins and peptides as shown in Figure 1 [16]. Moreover, α_s1_-casein and β-casein do not consist of cysteine or cystine, while α_s2_-casein and κ- casein consist of two half-cysteine residues that normally exist as homogeneous disulfide bonds.

#### 2.1.1. αs1-Casein

The α_s1_-casein consists of two hydrophobic regions containing all of the proline residues, which are separated by a polar region. The self-association of s1-casein is strongly influenced by its concentration, as well as the ionic strength, pH, and type of ion present in the medium; however, it is relatively temperature independent. It aggregates and precipitates at very low concentrations of Ca^2+^. A small number of peptides, sometimes called α-casein, are present in milk; these appear to originate from the proteolysis of α_s1_-casein [20].

#### 2.1.2. αs2-Casein

α_s2_-Casein has a unique dipolar structure, with negative charges concentrated near the N-terminus and positive charges concentrated near the C-terminus. The α_s2_-casein can strongly bind with calcium and is even more sensitive to precipitation by Ca^2+^ than α_s1_-casein. It self-associates at a neutral pH in the absence of Ca^2+^ [21].

#### 2.1.3. β-Casein

β-casein has a highly charged N-terminal region and a hydrophobic C-terminal region. The presence of a large number of Proline residues effectively prevents the formation of the extended helix. As a result, the β-casein molecule has a negatively charged head and an uncharged, essentially hydrophobic tail, similar to that of an anionic detergent. The self-association is temperature-dependent; it will form a large polymer at room temperature but not at 4 °C. It is less sensitive to calcium precipitation [22].

#### 2.1.4. κ-Casein

κ-casein is responsible for the stability of casein micelles, and it is very resistant to calcium precipitation. The Rennet cleavage at the Phe105-Met106 bond eliminates the stabilizing ability by leaving the hydrophobic portion, para-κ-casein, and a hydrophilic portion, called κ-casein glycomacropeptide [20].

### 2.2. Whey Proteins and Their Fractions

Whey proteins, also known as serum proteins, are globular proteins that contribute 20% of the total protein in bovine milk and practically all of the protein in whey. They are complex structured proteins with a high level of secondary and tertiary protein structures. Moreover, whey proteins are further fractionated in various important fractions, such as β-lactoglobulin (55%), α-lactalbumin (20%), immunoglobulins (13%), and Bovine serum albumin (BSA) (7%), which are minor proteins (5%) [23]. β-lactoglobulin, which is the major whey protein in bovine milk, is highly structured with α-helices or β-sheets, forming a packed globular structure with strong internal disulfide (S-S) bridges. The physical properties of whey proteins are shown in Table 2 [24].

#### 2.2.1. β-Lactoglobulin

This category, which includes eight genetic variations, accounts for around half of all whey proteins. There are two internal disulfide bonds and one free thiol group in β-lactoglobulin. The conformation has a lot of secondary structures, and is found as a noncovalently linked dimer in nature [17]. The dimers are further connected with octamers near the isoelectric point (pH 3.5 to 5.2), while they are separated from monomers at a pH below 3.4. [25].

#### 2.2.2. α-Lactalbumin

There are eight cysteine groups and four tryptophan residues in these proteins, all of which are involved in internal disulfide bonding. The secondary structure of α-lactalbumin is highly organized, while the tertiary structure is compact and spherical. The release of bound calcium is caused by thermal denaturation and a pH of less than 4.0 [26].

#### 2.2.3. Bovine Serum Albumin (BSA)

The molecular mass of BSA is 65 kDa; it contains 582 amino acid residues, 17 disulfides, and one sulfhydryl. All of the disulfides involve cysteines that are relatively close in the polypeptide chain, which is therefore organized in a series of relatively short loops. It binds Ca^2+^ and acts as a pH buffer. It generally has little or no effect in milk, though it may enhance lipase activity by binding metals and fatty acids [27].

#### 2.2.4. Immunoglobulins (IG)

Immunoglobulins are very complex proteins. There are five classes of IG, which are IGM, IGA, IGD, IGE, and IGG. Among these, IGM, IGG, and IGA are usually present in the milk. IGG has two subclasses, which are IGG1 and IGG2. There are disulfide links in IGG: two shorter (light) and long (heavy) polypeptide chains. IgA is made up of two of these units (eight chains) linked together by a secretory component (SC) and a junction (J) component, whereas IGM is made up of five four-chain units linked together. Each form of immunoglobulin has its own heavy and light chains [28].

#### 2.2.5. Minor Proteins

Milk contains several minor proteins, including about 60 indigenous enzymes, such as oxidoreductase, xanthine, lactoperoxidase, phosphatases, proteinase, and lipoprotein lipase. These are scientifically important. Most of the minor proteins have biological functions and probably play very significant roles [29].

## 3. Types of UHT Processing Systems for Milk

Generally, UHT processing of food is a combination of heat treatment with aseptic packing, which provides a shelf-life to a food product with minimal chemical damages as compared to in-container sterilized food products. Despite the fact that UHT milk has a shelf-life of up to 12 months, pasteurized milk has a larger sector of the milk market, as it is utilized as a convenience product [30]. UHT milk may require long-term stability, but in the latter scenario, the desired shelf life may be shorter than three months. To obtain ‘commercial sterility’, i.e., unlikely to grow bacteria in the product under typical storage conditions, UHT treatment in the range of 135–150 °C is used in combination with proper holding times [31]. In practice, the products are checked for sterility by incubating at 55 °C for 7 days or at 30 °C for 15 days, and testing for bacterial growth. UHT processing mainly consists of two types, which are direct and indirect heat treatments. The saturated steam is directly mixed with milk, and with the help of a vacuum flash vessel, the steam (added water) is removed from the milk in the direct heating method [26]. On the other hand, the heating medium, namely hot water or steam, is used to heats the milk indirectly by convection or conduction through a barrier in the form of a heat exchanger in the indirect method, as shown in Figure 1. The main difference between these two methods is that the effect on milk proteins can be differentiated in the temperature and time profiles. In the direct method of heating, the milk is heated and cooled very quickly from the sterilization temperature when compared with the indirect method of heating. Because of this reason, the direct method of heating would produce fewer chemical changes than the indirect method of heating. When the milk is processed by the direct method of UHT treatment, the formation of sediment occurs more as compared to the sedimentation that occurs in milk from indirect systems, and the rate of sedimentation is directly proportional to an increase in heat load and storage temperature [30]. Sterilization during aseptic packing of the milk is employed by the UHT process, and this operation enables UHT milk to be packaged without contamination, which provides a long shelf life to milk at an ambient temperature [31].

## 4. Effects of UHT Processing on Milk Proteins

There are many indications of milk proteins present in the UHT milk, and milk products are changed during processing and storage. Thermal treatments have a large range of impacts on milk components, depending on how intense they are, but they are usually associated with undesirable changes in product color, texture, and nutritional properties [32].

### 4.1. Effect of UHT Process on Casein Proteins

Denaturation of protein molecules occurs when it loses its native structure, by which it is strongly influencing the technological and biological functionality of that protein molecule. As discussed earlier, caseins do not contain a high level of secondary or tertiary structures [33]. High-temperature treatments exhibit little effect on the caseins, as evidenced by the fact that the bovine milk, which is heated at 140 °C for 20–30 min, does not form gelatin [34]. Such heat treatments will show other effects on casein proteins, such as dephosphorylation of amino acids [12]. Due to their higher levels of phosphorylation, α_s1_-casein, α_s2_-casein, β-casein, and κ-caseins can strongly bind with calcium, which makes caseins precipitate and aggregate, affecting their general stability, including their heat stability [35]. Generally, the heat stability of milk is affected by pH, at the native pH of 6.7, which is the maximum, and 6.9, which is the minimum [36]. The instability of milk to heat is not only caused by the casein micelles, but also by the denaturation of the whey proteins and their reaction with the micelles. This also results in changes in the calcium equilibrium [37]. As a function of pH, the solubilization of colloidal calcium phosphate and calcium precipitation also occurs at above 100 °C [20], though these changes do not disrupt the overall basic structure of casein micelles or colloidal destabilization.

#### Dissociation of Casein Proteins from the Casein Micelles

**Figure 2:**
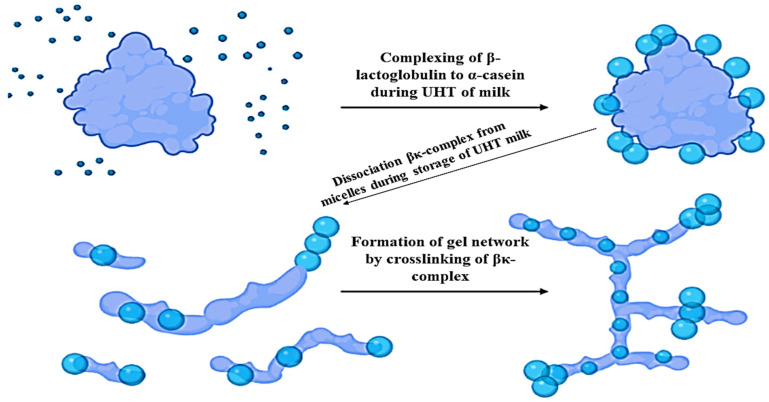
Effect of UHT process on structural chemistry of casein proteins [36]. According to the proposed mechanism, UHT milk gelation is due to specific k-casein proteolysis. Dissociation of β- k-casein complex occurs, which leads to gel-like structure formation by crosslinking of β- k-casein complex.

The pH-dependent dissociation of casein proteins from the micelles can be caused by the heat treatment of milk [38]. The process of dissociation is dependent on the duration of heating, temperature of heating, and the composition of the milk. As the pH increases, the dissociation of caseins also increases. At a pH of 7.1, significant levels of caseins are dissociated from the micelles. The composition of dissociated casein is dependent on the heating temperatures. At a temperature of 70 °C, all of the caseins are in a state of dissociated protein. The proportions are altered from natively found, with higher levels of κ-casein, β-casein about the same level, and lower levels of α_s_-casein than that of the whole casein protein, as shown in Figure 2 [36]. Moreover, during the UHT of milk, the complexing of the α_s1_-casein takes place; however, the gel network is formed by crosslinking of β- and κ-casein. Therefore, the dissociated level of casein continues to increase, whereas α_s_-casein and β-casein levels decrease gradually as the temperature is raised above 70 °C, so that the κ-casein is found in the milk serum at a pH of 7.1 and higher temperatures [38]. The dissociated α_s_-casein and β-casein levels are increased due to decreasing the pH to 6.0, and dissociated κ-casein levels are decreased; however, lowering the pH further increases the level of all caseins dissociating [36].

### 4.2. Effect of UHT Process on Whey Proteins

**Figure 3:**
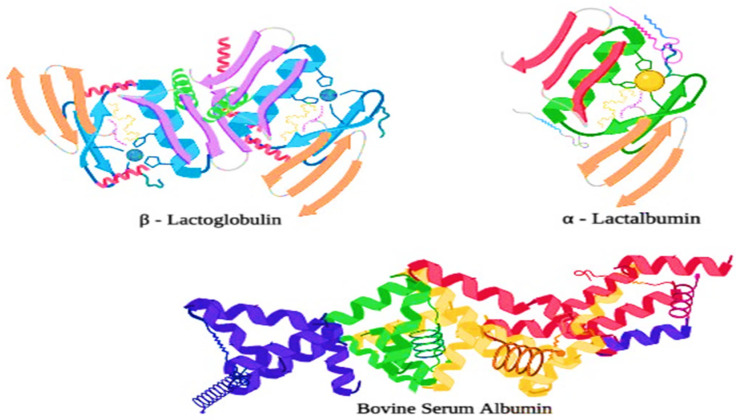
The polymeric structure of whey proteins and their fractions. β-lg belongs to the lipocalin family of proteins, which all contain a β-barrel composed of antiparallel β-sheets. In β-lg, each β sheet has one hydrophobic side and one hydrophilic side. The two hydrophobic sides face each other, creating a hydrophobic cavity. The α-lactalbumin (α-la) is a globular 123-amino acid, 14.2 kDa protein. A, B, and C are considered as genetic variants, and BSA is a 582-amino acid residue protein. The structure consists of three domains stabilized by 17 disulfide bonds and one free thiol group [16].

Most of the major whey proteins are highly ordered globular proteins, and thus they are very heat sensitive as they are liable to structural damage at the time of processing treatments in the dairy industries (e.g., high-pressure homogenization, and heat treatments). Generally, whey proteins tend to unfold from their original structure, revealing amino acids such as cysteine, which contains a free sulfhydryl (-SH) group when they are exposed to temperatures greater than 65 °C [39]. Moreover, the exposed free sulfhydryl group is now readily available and highly reactive, allowing other denatured whey proteins and proteins that contain the exposed sulfhydryl groups (e.g., κ-casein) to interact and aggregate to form a new (S-S) disulfide bond [35]. Whey protein fractions are composed of several proteins such as lactoferrin (LTF), α-lactalbumin (α-LA), β-lactoglobulin (β-LG) a small amount of lactoperoxidase (LP), bovine serum albumin (BSA), and immunoglobulin (IG). The structures of α-LA, β-LG, and BSA have been shown in Figure 3. Moreover, β-LG has two disulfide bonds which maintain the structural integrity during the heat treatment and hydrolysis treatment, and one free cysteine group. 

**Figure 4:**
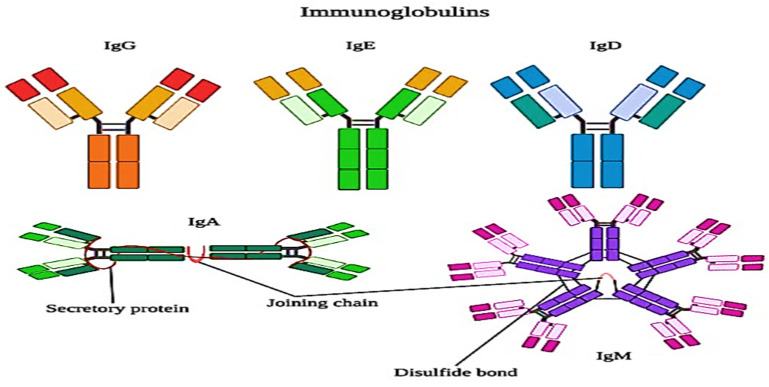
The polymeric structure of different types of immunoglobulins present in milk.

Whey proteins are more susceptible to heat denaturation, and their susceptibility depends upon the structural chemistry of the proteins. Immunoglobulins are the most heat-sensitive whey proteins, which are followed by bovine serum albumin (BSA), β-lactoglobulin (β-Lg), α-lactalbumin (α-La), and proteose peptones, though the last are not affected by heat. Mostly, the UHT treatments denature the immunoglobulins and most of the bovine serum albumin (BSA) because these proteins are present in lower concentrations than β-lactoglobulin and α-lactalbumin. The structure of immunoglobulins is explained in Figure 4. The effects of denaturation and interaction with other proteins are minor as compared to β-lactoglobulin and α-lactalbumin, whereas, in the milk, the denaturation of whey proteins is dominated by the denaturation of β-lactoglobulin, which is about 50% of the whey proteins. However, α-lactalbumin is also important [39].

The immunoglobulin (Ig) fraction is a complex heterogeneous mixture of large glycoproteins which possess antibody activity. With molecular weights ranging from 180,000–900,000 Da, there are four classes of immunoglobulins in bovine milk: IgM, IgA, IgE, and IgG [8]. The latter class is subdivided into IgG1 and IgG2, and is found in milk in monomeric form while the others are found in polymeric form. IgG1 is the main Ig class in bovine milk, representing about 80% of total Ig.

#### The Process of Whey Protein Denaturation

β-lactoglobulin is a globular protein that occurs as a dimer, with a higher molecular weight of 36 kDa at the normal pH of the milk. When heat is applied to -Lg, it unfolds and separates into two monomers, each of which has a disulfide bond, and the reactive free sulfhydryl group that was buried in the native structure. At a temperature of 70 °C, the process of denaturation is irreversible, because exposed monomer forms the disulfide bonds with other β-lactoglobulin and α-lactalbumin molecules, and also with the κ-casein through the interactions of thiol-disulfide. However, due to the interaction with the other cysteine-containing casein molecules, α_s2_-casein may also occur. Similarly, the interactions contain hydrophobic bonding through the hydrophobic parts of β-lactoglobulin molecules that are exposed by the process of denaturation [16]. Furthermore, α-lactalbumin has no free sulfhydryl group, but it reacts with β-lactoglobulin through the interaction of thiol-disulfide. At higher temperatures, the reactive free sulfhydryl groups are released by the disruption of one of the disulfide bonds, which can initiate and spread the irreversible aggregation in the same method to the naturally occurring free sulfhydryl in β-lactoglobulin. Bovine serum albumin contains a free sulfhydryl group that could take part in the intermolecular interactions, whereas the mixture of β-lactoglobulin and BSA are heated for 15 min at a temperature of 75 °C. Then, the BSA forms the homopolymers [40]. At higher temperatures, the bovine serum albumin remains inactive and aggregated during heating to 75 °C, at which point β-lactoglobulin forms the intermolecular aggregates. Also, during the intermolecular complex formation in the UHT milk, the bovine serum albumin may be involved to a limited extent.

In addition, the denatured whey proteins can interact with casein fractions and form a composite of whey protein and κ-casein either in the serum phase or on the surface of the casein micelle [16]. This composite formation may prevent the dissociation of α_s_-casein and β-caseins with increasing temperature [41], and thus lowers their levels in the serum of UHT milk when compared with pasteurized milk. The dissociation of κ-casein from the casein micelle is an important factor related to the denaturation of β-lactoglobulin. During the UHT processing of skim milk (total solids 12–15%), the total percentage of κ-casein from the casein micelle at pH 6.6 was 30–60% [37]. Therefore, κ-casein interacts with denatured β-lactoglobulin in either part of the casein micelle or serum phase. The release of κ-casein from the casein micelle led to the denaturation of β-lactoglobulin [41]. Another important element is determining how much denatured whey protein is bound to κ-casein in the casein micelle and how much is associated with κ-casein in the serum phase [42]. When the milk is heated at a pH of 6.5, the denatured whey proteins are favorably attached to the casein micelle, whereas, at a greater pH > 6.8, most of the whey proteins or serum proteins are attached to the κ-casein, and they are found in the serum. The amount of attached whey proteins or serum proteins is highly sensitive to minor changes in the pH. The relative amounts of β-Lg and α-La that become attached to the micelle are also influenced by the heating process. Rapid heating, such as that used in indirect UHT processing, generates a large β-Lg: α-La ratio, but slower heating, such as that used in indirect UHT treatments, produces a lower ratio, indicating that more α-La and less β-Lg become attached [38,43]. 

## 5. Changes in the Protein during Processing and Storage

Milk and milk products are the richest sources of proteins for the human diet [44]. Moreover, several different thermal treatments, such as ultrahigh temperature (UHT) or sterilization (140 °C for 3–4 s), may be used during milk processing to increase the product’s shelf life at room temperature while also ensuring its microbiological safety [44]. During the processing of a food product containing milk, proteins may cause protein unfolding, hydrophobic group exposure, aggregation, and flocculation. All of these changes may have a positive or negative impact on their techno-functional properties [45]. 

### 5.1. Proteolysis

Proteolysis occurs in many UHT products, but it can be minimized or prevented by properly selecting the quality of the raw milk and carefully choosing the processing conditions. Although some non-enzymatic proteolysis can occur during the heat treatment of the milk, this is usually produced by proteases. Exogenous or indigenous proteases can produce enzymatic proteolysis [46,47]. In the exogenous enzymes, heat resistant proteases are produced by the psychrotrophic bacteria in the raw milk, particularly pseudomonas bacterial species, which are the most important. However, proteases produced by the spore-forming bacteria, like Bacillus and Bacillus-like species, are also involved [48].

### 5.2. Age Gelation

The reduction in the shelf life of UHT milk is caused mainly due to the effect of age gelation. It is well known that proteolysis by plasmin or bacterial proteases is the major initiator of gelation [49]. The process of gelation is observed in the milk in which the proteolysis did not appear to be a factor. This type of gelation is described as physicochemical [50]. This physicochemical gelation is likely to occur in UHT milk with a high solids content, i.e., concentrated milk, although the nonconcentrated milk has also been reported, where the gelation occurs in the milk after long-term storage [51]. Several studies indicated the involvement of proteolysis in age gelation. The proteolysis is caused by both bacterial proteases and plasmin activity, which has been proven by many authors [50,52]. However, most of the analyses on proteases were not able to find the levels that can cause gelation after the long-term storage of the milk. The protease assays, which are involving long incubation times, detect low protease activities [52]. Australian scientists have developed the cyber tongue method, which is a biosensor-based method, and the biosensor uses a Bioluminescence Resonance Energy Transfer (BRET) transduction system, that can detect the proteases in the milk at concentrations within a few minutes to the industry [49].

#### Process of Age Gelation

As indicated earlier, the process of proteolysis occurs either through plasmin activity or bacterial proteases as the main cause of age gelation in milk during storage, but the gelation is also caused by physicochemical changes. The proteolysis-induced age gelation generally appears through a three-phase process. In the first phase, during the heat treatment, the interaction of β-lactoglobulin with κ-casein on the surface of the casein micelle and in the serum phase forms a complex of β-Lg and κ-casein [4]. The electron microscope is used to show how the tendrils of β-Lg and κ-casein complex attach to the micelles [53]. In the second phase, the proteolysis of the casein proteins in the micelle, which splits the peptide bonds that connect the κ-casein to the casein micelle, enables the release of the complex of β-Lg and κ-casein tendrils into the serum. In the third phase, the formation of gel from the complex of β-Lg and κ-casein in the serum occurs, when the complex concentration exceeds a critical level. 

### 5.3. Temperature-Time Conditions of Heat Treatment and Storage

The temperature-time condition in the process of the sterilization holding tube is used to minimize the proteolysis and age gelation processes [53]. For example, increasing the temperatures from 145 °C to 150 °C at a constant time of four seconds reduces proteolysis and also the bitterness in UHT milk, although increasing the severity of the temperature has a major effect on the flavor of the product.

### 5.4. Sedimentation

Sedimentation is a major problem that occurs in UHT milk, and it is defined as a substance that settles to the bottom of the container compactly. Generally, a small amount of sediment is present in the UHT milk. The amount of sediment from the UHT processing of good quality milk should be less than 0.5%. The amount of sediment increases with storage time [54]. The sediment in the UHT milk largely consists of aggregated κ-casein and depleted casein micelles, with minor amounts of whey proteins and κ-casein [53,54,55]. Grewal et al. [56] reported that changes to FTIR were linked with the confirmation of fat in the whole milk samples as stored for 14 days at 40 °C–50 °C. This indicates the formation of an intermolecular β-sheet of proteins, which shows interactions of protein-lipids and aggregation. The sediment from these whole milk samples contains fat, which proves the interaction of protein-lipid in the sedimentation process. In this regard, Boumpa et al. [55] reported that, regarding the sediment in some UHT mineral-fortified milk, the fortification is done with the insoluble calcium salts, such as calcium citrate or calcium carbonate, which creates a high proportion of mineral salts as the sediments. When the pH of the milk is reduced, the risk of sedimentation is high and excessive at pH < 6.62 for milk at its natural concentration, or below pH 6.55 for two times concentrated milk [57]. The addition of calcium salts to milk also increases the sedimentation level. At constant pH, sedimentation in the milk could be increased by increasing the Ca^2+^ levels and decreased by decreasing the Ca^2+^ levels. In the same way, at constant Ca^2+^ levels, sedimentation in the milk increased by decreasing the pH of milk, and the sediment level could be decreased by increasing the pH of the milk. This exhibits the clear relationship between pH and ionic calcium on sedimentation [17].

### 5.5. Lactosylation

Maillard’s reaction is a chemical reaction between amino acids and reducing sugars that gives browned food its distinctive flavor. In the milk, this early reaction is generally lactosylation, the reaction between the ε-amino group of lysine and, to a lesser amount, methionine, tryptophan, arginine, histidine, and lactose [7]. The major product, protein-bound Schiff’s base lactosyl is formed with lysine, but this readily undergoes an Amadori rearrangement, which is highly significant to producing more stable protein-bound lactulosyl lysine (ε-N-deoxylactulosyl-L-lysine). The lysine in the Amadori product of the rearrangement is biologically unavailable, and the lactosylated lysine delays proteolysis of the protein molecule with the help of digestive proteases [58]. This affects the nutritional quality of the milk because of lysine blockage. However, a reduction in nutritive value raises concerns with infant formulas, including UHT-processed formulas. Infant formulas usually have a higher amount of whey proteins to casein [59], and the whey proteins are highly rich in lysine content at about 12%. Thus, the product still contains an abundant amount of available lysine. The process of lactosylation can be observed by methods based on mass spectrometry [60]. This method was used to observe the different forms of lactosylated whey proteins and caseins, as each lactose adds 324 Da to the molecular weight. These lactosylated forms can be observed not only in the mass spectrographs, but also in 2-D electrophoresis gels, in which they are easily seen for β-lactoglobulin and α-lactalbumin. More molecules of lactose are attached to the milk proteins with the increase in the intensity of heat treatment and time and temperature of storage conditions [60]. There is a significant decrease in the value of pH when Millard browning occurs in UHT milk. This is due to the natural formation of formic acid and acetic acid, which are products of the Maillard reaction. In conclusion. during heat treatment and following storage of UHT milk, the Maillard reaction is one of the major quality deterioration factors. The formation of the Amadori product e-lactulosyllysine between lactose and the free ε-amino group of lysine results in a decreased nutritional value of the milk, as the lysine will no longer be bioavailable, and extensive lactosylation can also reduce the digestibility of proteins.

### 5.6. Deamidation

Deamidation is the hydrolysis of amide groups in glutamine (Gln) and asparagine (Asn) residues to ammonia, as well as glutamic acid (Glu) and aspartic acid (Asp), respectively. During normal UHT processing, non-enzymatic deamidation occurs to only a small degree, but it occurs significantly during the storage of UHT milk [17,60]. The process of deamidation was increased with temperature and time of storage. The residues of asparagine are adjacent to glycines, exhibiting higher rates of deamidation, whereas deamidation of Asn occurs actively compared to glutamine. The deamidation of α_s1_-casein can be observed with the use of 2-D electrophoresis, which shows one spot for this casein at the manufacture. During the storage conditions, this spot splits into multiple spots with approximately similar molecular weights. However, each new spot shows the loss of ammonia and one negative charge [61]. When the UHT milk samples are stored at 40 °C for 60 days, up to four new spots have appeared, and each spot represents more than one molecular species with the same charge. Some of the sites of deamidation are Glm24, Asn32, Asn129, and Asn205. The Asn 63 is the site for the deamidation of β-LgA and β-LgB. The proteins in a shelf-stable acidic whey protein beverage, which are produced by heating at a temperature of 120 °C for 20 s, are deamidated when they are stored for 12 months [31]. Le et al. [62] suggested that deamidation may increase the susceptibility of proteins and peptides to acid hydrolysis by converting asparagine (Asn) into aspartic acid (Asp), which makes an extra hydrolysis site available. This acid hydrolysis site is beneficial during the storage of these beverages, by reducing the formation of caseinomacropeptide (CMP)-rich aggregates. Likewise, deamidation of proteins can increase their heat stability, preserve their nutritive value through reducing the blockage of lysines, improve some physical functional properties, and reduce the amount of fouling deposit formation [63]. 

## 6. Challenges for UHT-Treated Milk Consumption

The most common spoilage signs of UHT milk causing consumer complaints and product recalls with high economic costs are sedimentation, gelation, and fat separation [64]. The accessibility of UHT milk may have a great impact on both food security policies for developing countries and strategies to limit the worldwide wastage of food. Implementing the high-quality production of UHT milk may extend the storage stability of the milk by up to 10–12 months, which represents a challenging issue to improve the market in new areas, where the consumption of milk may potentially grow. To delay fat creaming in UHT milk, some of the technologies include ultrasonics; membrane emulsification; macro fluidization; high-speed mixing [65]; ultra-high-pressure homogenization (UHPH), which is carried out up to 400–600 MPa; and high-pressure homogenization (HPH), which reaches 200 MPa [66,67]. The initial microfiltration of milk delays both the gelation and sedimentation significantly, while the UHT treatment was conducted at low heating temperatures. The presence of fat in the milk may either favor or compete with the protein interactions. By using double homogenization performed at 250 MPa and 400 MPa as a pre-UHT and post-UHT treatment, fat creaming in UHT milk was effectively prevented for 18 months. Therefore, the combination of ultra-high-pressure homogenization (UHPH) and microfiltration to the UHT milk was a very effective approach to guarantee the shelf-storage stability of UHT milk at room temperature for up to 12 months without any increase in the severity of the sterilization conditions [68]. These processing technological approaches can be implemented to extend the shelf-storage stability of UHT milk.

## 7. Conclusions

UHT-treated milk is a successful method to extend the shelf-life of milk and its products. There are some cases when the shelf-life of the product is decreased due to some unacceptable and permanent changes to the products at the time of storage. The serious problems of these are sedimentation, creaming, and age gelation. The gelation can be easily controlled by proper heat treatments to complete the inactivation of enzyme activity for plasmin-induced age gelation, or by using good quality milk with good manufacturing practices (GMP), which consequently avoids age gelation through exogenous enzymes. Interestingly, some recent studies revealed that the composition of aggregated material in UHT milk is the same as that of gelled material from the physicochemical mechanism of age gelation. This involves the aggregation of κ-casein-depleted casein micelles. This tendency for milk can form sediment or gel, which depends on the pH or ionic calcium levels during manufacturing and storage. As this aggregation of κ-casein-depleted casein micelles is slow, the gelation occurs slightly as compared to the formation of a compact sediment. In many cases, this gelation occurs more quickly than the proposed shelf-life of the milk. There are many indications of milk proteins present in the UHT milk, and milk products are altered during processing and storage. 

## Figures and Tables

**Table 1:** Characterization of caseins based on molecular mass, amino acid numbers, proline, and cysteine residues.

Milk Proteins (Caseins)	Molecular Mass	Amino Acids	Proline Residues	Cysteine Residues	PO_4_ Groups	Concentration (g/L)	Glycoproteins	References
α_s1_-casein	23,164	199	17	0	8	10	No	[2,17]
α_s2_-casein	25,388	207	10	2	10–13	2.6	No	[2,17]
β-casein	23,983	209	35	0	5	9.3	No	[2,17]
κ-casein	19,038	169	20	2	1	10.3	Yes	[2,17]

**Table 2:** Physical properties, molecular weight, and concentration of whey proteins in milk.

Milk Proteins (Whey Proteins)	% of Whey Protein	Isoelectric Point (pI)	Concentration (g/L)	Molecular Weight (kDa)	References
β-lactoglobulin	55–65	5.35–5.49	3.3	18.4	[2,24]
α-lactalbumin	15–25	4.2–4.5	1.2	14.2	[2,24]
Bovine serum albumin (BSA)	5–6	5.1	0.3	66.3	[2,24]
Immunoglobulins	10–15	5.5–8.3	0.5	80–900	[2,24]
Proteose peptones	10–20	5.1–6.0	0.2	4.1–80	[2,24]

## Data Availability

Data sharing is not applicable to this article.

## References

[1] Figueiredo B., Dias C.C., Brandão S., Canário C., Nunes-Costa R. (2013). Breastfeeding and postpartum depression: Review of the state of the art. J. Pediatr..

[2] Fox P.F., Uniacke-Lowe T., McSweeney P.L.H., O’Mahony J.A. (2015). Milk proteins. Dairy Chemistry and Biochemistry.

[3] Moughan P.J. (2014). Milk Proteins—A Cornucopia for Developing Functional Foods. Milk Proteins.

[4] Anema S.G. (2017). Storage stability and age gelation of reconstituted ultra-high temperature skim milk. Int. Dairy J..

[5] Dupont D., Tomé D. (2020). Milk proteins: Digestion and absorption in the gastrointestinal tract. Milk Proteins.

[6] Lewis M., Grandison A., Lin M., Tsioulpas A. (2011). Ionic calcium and pH as predictors of stability of milk to UHT processing. Milchwissenschaft.

[7] Rauh V.M., Johansen L.B., Bakman M., Ipsen R., Paulsson M., Larsen L.B., Hammershøj M. (2015). Protein lactosylation in UHT milk during storage measured by liquid chromatography–mass spectrometry and quantification of furosine. Int. J. Dairy Technol..

[8] Bimbo F., Bonanno A., Liu X., Viscecchia R. (2016). Hedonic analysis of the price of UHT-treated milk in Italy. J. Dairy Sci..

[9] Decimo M., Morandi S., Silvetti T., Brasca M. (2014). Characterization of gram-negative psychrotrophic bacteria isolated from Italian bulk tank milk. J. Food Sci..

[10] Chavan R.S., Chavan S.R., Khedkar C.D., Jana A.H. (2011). UHT milk processing and effect of plasmin activity on shelf life: A review. Compr. Rev. Food Sci. Food Saf..

[11] Gaucher I., Mollé D., Gagnaire V., Gaucheron F. (2008). Effects of storage temperature on physico-chemical characteristics of semi-skimmed UHT milk. Food Hydrocoll..

[12] Orcajo J., de Marañon I.M., Lavilla M. (2015). Antigenic response of bovine β-lactoglobulin influenced by ultra-high pressure treatment in combination with high temperature. Clin. Transl. Allergy.

[13] Pizzano R., Manzo C., Adalgisa Nicolai M., Addeo F. (2012). Occurrence of major whey proteins in the pH 4.6 insoluble protein fraction from UHT-treated milk. J. Agric. Food Chem..

[14] Agarwal A., Pathera A.K., Kaushik R., Kumar N., Dhull S.B., Arora S., Chawla P. (2020). Succinylation of milk proteins: Influence on micronutrient binding and functional indices. Trends Food Sci. Technol..

[15] Karlsson M.A., Langton M., Innings F., Malmgren B., Höjer A., Wikström M., Lundh Å. (2019). Changes in stability and shelf-life of ultra-high temperature treated milk during long term storage at different temperatures. Heliyon.

[16] Walstra P. (1999). Casein sub-micelles: Do they exist?. Int. Dairy J..

[17] Hazlett R., Schmidmeier C., O’Mahony J.A. (2018). Milk Proteins. Encyclopedia of Food Chemistry.

[18] Morgan A.A., Rubenstein E. (2013). Proline: The distribution, frequency, positioning, and common functional roles of proline and polyproline sequences in the human proteome. PLoS ONE.

[19] Phadungath C. (2005). Casein micelle structure: A concise review. Songklanakarin J. Sci. Technol..

[20] Fox P.F., Uniacke-Lowe T., McSweeney P.L.H., O’Mahony J.A. (2015). Heat-induced changes in milk. Dairy Chemistry and Biochemistry.

[21] Huppertz T. (2013). Chemistry of the caseins. Advanced Dairy Chemistry.

[22] Huppertz T., Fox P.F., Kelly A.L. (2004). High pressure treatment of bovine milk: Effects on casein micelles and whey proteins. J. Dairy Res..

[23] Boland M. (2011). Whey proteins. Handbook of Food Proteins.

[24] O’mahony J.A., Fox P.F. (2013). Milk proteins: Introduction and historical aspects. Advanced Dairy Chemistry.

[25] Sawyer L. (2013). β-Lactoglobulin. Advanced Dairy Chemistry.

[26] Brew K. (2013). α-Lactalbumin. Advanced Dairy Chemistry.

[27] Nicholson J.P., Wolmarans M.R., Park G.R. (2000). The role of albumin in critical illness. Br. J. Anaesth..

[28] Hurley W.L., Theil P.K. (2013). Immunoglobulins 9 Secretions. Advanced Dairy Chemistry, Volume 1A: Proteins: Basic Aspects.

[29] Wynn P.C., Sheehy P.A. (2013). Minor proteins, including growth factors. Advanced Dairy Chemistry.

[30] Deeth H.C., Lewis M.J. (2017). High Temperature Processing of Milk and Milk Products.

[31] Robertson G.L., John W.F., Patrick E.F., Paul L.H. (2011). Heat Treatment of Milk: Ultra-High Tempterature Treatment (UHT: Aspect Packaging. Encyclopedia of Dairy Sciences.

[32] Deeth H.C. (2020). The effect of UHT processing and storage on milk proteins. Milk Proteins.

[33] Singh H., Latham J.M. (1993). Heat stability of milk: Aggregation and dissociation of protein at ultra-high temperatures. Int. Dairy J..

[34] Anema S.G., Li Y. (2000). Further studies on the heat-induced, pH-dependent dissociation of casein from the micelles in reconstituted skim milk. LWT Food Sci. Technol..

[35] Singh H., Creamer L.K. (1991). Influence of concentration of milk solids on the dissociation of micellar κ-casein on heating reconstituted milk at 120 °C. J. Dairy Res..

[36] Dumpler J., Wohlschläger H., Kulozik U. (2017). Dissociation and coagulation of caseins and whey proteins in concentrated skim milk heated by direct steam injection. Dairy Sci. Technol..

[37] Wijayanti H.B., Bansal N., Deeth H.C. (2014). Stability of whey proteins during thermal processing: A review. Compr. Rev. Food Sci. Food Saf..

[38] Oldfield D.J., Singh H., Taylor M.W. (1998). Association of β-lactoglobulin and β-lactalbumin with the casein micelles in skim milk heated in an ultra-high temperature plant. Int. Dairy J..

[39] Wijayanti H.B., Brodkorb A., Hogan S.A., Murphy E.G. (2019). Thermal denaturation, aggregation, and methods of prevention. Whey Proteins.

[40] Havea P., Singh H., Creamer L.K. (2001). Characterization of heat-induced aggregates of beta-lactoglobulin, alpha-lactalbumin and bovine serum albumin in a whey protein concentrate environment. J. Dairy Res..

[41] Anema S.G. (2008). On heating milk, the dissociation of [kappa]-casein from the casein micelles can precede interactions with the denatured whey proteins. J. Dairy Res..

[42] Anema S.G., Li Y. (2003). Effect of pH on the association of denatured whey proteins with casein micelles in heated reconstituted skim milk. J. Agric. Food Chem..

[43] Anema S.G. (2009). Effect of milk solids concentration on the pH, soluble calcium and soluble phosphate levels of milk during heating. Dairy Sci. Technol..

[44] Borad S.G., Kumar A., Singh A.K. (2017). Effect of processing on nutritive values of milk protein. Crit. Rev. Food Sci. Nutr..

[45] Qi P.X., Ren D., Xiao Y., Tomasula P.M. (2015). Effect of homogenization and pasteurization on the structure and stability of whey protein in milk. J. Dairy Sci..

[46] Raikos V. (2010). Effect of heat treatment on milk protein functionality at emulsion interfaces. A review. Food Hydrocoll..

[47] Gaucher I., Molle D., Gagnaire V., Leonil J., Rousseau F., Gaucheron F. (2009). Destabilization of commercial UHT milks: Proteolysis and changes in milk particles. Milchwissenschaft.

[48] Datta N., Deeth H.C. (2003). Diagnosing the cause of proteolysis in UHT milk. LWT Food Sci. Technol..

[49] Deeth H., Lewis M. (2016). Protein stability in sterilised milk and milk products. Advanced Dairy Chemistry.

[50] Datta N., Deeth H.C. (2001). Age gelation of UHT milk—A review. Food Bioprod. Process..

[51] Mitchell G.E., Ewings K.N. (1985). Quantification of bacterial proteolysis causing gelation in UHT-treated milk. N. Z. J. Dairy Sci. Technol..

[52] Button P.D., Roginski H., Deeth H.C., Craven H.M. (2011). Improved shelf-life estimation of UHT milk by prediction of proteolysis. J. Food Qual..

[53] Malmgren B., Ardö Y., Langton M., Altskär A., Bremer M.G., Dejmek P., Paulsson M. (2017). Changes in proteins, physical stability and structure in directly heated UHT milk during storage at different temperatures. Int. Dairy J..

[54] Stoeckel M., Lidolt M., Achberger V., Glück C., Krewinkel M., Stressler T., Hinrichs J. (2016). Growth of Pseudomonas weihenstephanensis, Pseudomonas proteolytica and Pseudomonas sp. in raw milk: Impact of residual heat-stable enzyme activity on stability of UHT milk during shelf-life. Int. Dairy J..

[55] Boumpa T., Tsioulpas A., Grandison A.S., Lewis M.J. (2008). Effects of phosphates and citrates on sediment formation in UHT goats’ milk. J. Dairy Res..

[56] Grewal M.K., Chandrapala J., Donkor O., Apostolopoulos V., Stojanovska L., Vasiljevic T. (2017). Fourier transform infrared spectroscopy analysis of physicochemical changes in UHT milk during accelerated storage. Int. Dairy J..

[57] Gaur V., Schalk J., Anema S.G. (2018). Sedimentation in UHT milk. Int. Dairy J..

[58] Mehta B.M., Deeth H.C. (2016). Blocked lysine in dairy products: Formation, occurrence, analysis, and nutritional implications. Compr. Rev. Food Sci. Food Saf..

[59] Fenelon M.A., Hickey R.M., Buggy A., McCarthy N., Murphy E.G. (2019). Whey proteins in infant formula. Whey Proteins.

[60] Holland J.W., Gupta R., Deeth H.C., Alewood P.F. (2011). Proteomic analysis of temperature-dependent changes in stored UHT milk. J. Agric. Food Chem..

[61] Holland J.W., Gupta R., Deeth H.C., Alewood P.F. (2012). UHT milk contains multiple forms of αS1-casein that undergo degradative changes during storage. Food Chem..

[62] Le T.T., Nielsen S.D., Villumsen N.S., Kristiansen G.H., Nielsen L.R., Nielsen S.B., Larsen L. (2016). BUsing proteomics to characterise storage-induced aggregates in acidic whey protein isolate drinks. Int. Dairy J..

[63] Miwa N., Yokoyama K., Wakabayashi H., Nio N. (2010). Effect of deamidation by protein-glutaminase on physicochemical and functional properties of skim milk. Int. Dairy J..

[64] Cattaneo S., Masotti F., Pellegrino L. (2008). Effects of overprocessing on heat damage of UHT milk. Eur. Food Res. Technol..

[65] Huppertz T. (2011). Homogenization of Milk|Other Types of Homogenizer (High-Speed Mixing, Ultrasonics, Microfluidizers, Membrane Emulsification). Encyclopedia of Dairy Sciences.

[66] Dumay E., Chevalier-Lucia D., Picart-Palmade L., Benzaria A., Gràcia-Julià A., Blayo C. (2013). Technological aspects and potential applications of (ultra) high-pressure homogenisation. Trends Food Sci. Technol..

[67] Schlender M., Minke K., Spiegel B., Schuchmann H.P. (2015). High-pressure double stage homogenization processes: Influences of plant setup on oil droplet size. Chem. Eng. Sci..

[68] D’Incecco P., Rosi V., Cabassi G., Hogenboom J.A., Pellegrino L. (2018). Microfiltration and ultra-high-pressure homogenization for extending the shelf-storage stability of UHT milk. Food Res. Int..

